# Cellular Interplay between Cardiomyocytes and Nonmyocytes in Cardiac Remodeling

**DOI:** 10.4061/2011/535241

**Published:** 2011-09-18

**Authors:** Norifumi Takeda, Ichiro Manabe

**Affiliations:** ^1^Department of Cell and Developmental Biology and Penn Cardiovascular Institute, Perelman School of Medicine at the University of Pennsylvania, Philadelphia, PA 19104, USA; ^2^Department of Cardiovascular Medicine, Graduate School of Medicine, University of Tokyo, 7-3-1 Hongo, Bunkyo, Tokyo 113-8655, Japan; ^3^Global COE Program, Graduate School of Medicine, University of Tokyo, Tokyo 113-8655, Japan

## Abstract

Cardiac hypertrophy entails complex structural remodeling involving rearrangement of muscle fibers, interstitial fibrosis, accumulation of extracellular matrix, and angiogenesis. Many of the processes underlying cardiac remodeling have features in common with chronic inflammatory processes. During these processes, nonmyocytes, such as endothelial cells, fibroblasts, and immune cells, residing in or infiltrating into the myocardial interstitium play active roles. This paper mainly addresses the functional roles of nonmyocytes during cardiac remodeling. In particular, we focus on the communication between cardiomyocytes and nonmyocytes through direct cell-cell interactions and autocrine/paracrine-mediated pathways.

## 1. Introduction

Cardiac hypertrophy is an essential adaptive process, through which the heart responds to various mechanophysical, metabolic, and genetic stresses. On the other hand, the hypertrophy induced by sustained overload eventually leads to contractile dysfunction and heart failure. Cardiac hypertrophy involves cellular and molecular events within both cardiomyocytes and nonmyocytes. Cardiomyocytes show phenotypic modification that results in cellular hypertrophy accompanied by reexpression of several fetal genes, abnormal Ca^2+^ handling, oxidative stress and mitochondrial DNA damage, and cardiomyocyte death due to necrosis or apoptosis [1]. In addition to cardiomyocytes, the myocardium contains a variety of nonmyocytes, including vascular endothelial, and smooth muscle cells, fibroblasts and immune cells, which all appear to be crucially involved in the myocardial response to external and internal stress [2, 3]. During cardiac hypertrophy and the progression to heart failure, the myocardium exhibits complex structural remodeling involving rearrangement of muscle fibers, fibrosis, accumulation of extracellular matrix (ECM), cellular death, and angiogenesis [4]. Many of the processes underlying these phenomena are also seen in chronic inflammatory diseases and are mediated by cellular interactions among cardiomyocytes and nonmyocytes. In this paper, we will focus on the functional roles of nonmyocytes and the cellular communication ongoing during the development of cardiac hypertrophy and heart failure under noninfectious and noninfarction conditions, such as pressure overload.

## 2. Fibroblasts

Cardiac fibroblasts are critically involved in the development of cardiac fibrosis [4, 5]. They can produce a wide variety of ECM proteins, including interstitial collagens, proteoglycans, glycoproteins, and proteases [6]. Morphologically, fibroblasts are flat, spindle-shaped cells with multiple processes sprouted from the cell body, which lacks a basement membrane [7]. Fibroblasts play central roles in two types of fibrosis: reparative and reactive. Reparative (replacement) fibrosis or scarring accompanies cardiomyocyte death. Reactive fibrosis appears as “interstitial” or “perivascular” fibrosis and does not directly associate with cardiomyocyte death [8, 9].

Increases in fibrosis result in mechanical stiffness and cardiac diastolic dysfunction [10]. In addition, by forming a barrier between cardiomyocytes, fibrosis can impair the electrical coupling of cardiomyocytes, leading to cardiac systolic dysfunction [11]. Moreover, perivascular fibrosis can increase oxygen and nutrient diffusion distances, leading to pathological remodeling [12]. Thus, fibrosis profoundly affects cardiomyocyte metabolism and performance, and ultimately ventricular function [13]. However, the functions of fibroblasts are not limited to producing ECM. Cardiac fibroblasts interact with other cell types, most notably cardiomyocytes. This interaction may be direct via physical contacts or indirect via paracrine factors. Thus fibroblasts are involved in much more than deposition of collagen [4, 7, 14].

In response to external stress, fibroblasts change their phenotype and become myofibroblasts [15, 16], which express several smooth muscle (SM) markers, including SM *α*-actin, SM22*α*, SMemb/nonmuscle myosin heavy chain-B, and tropomyosin [16–18]. However, more stringent SM markers (e.g., SM myosin heavy chains) are not expressed in myofibroblasts [19]. With the exception of heart valve leaflets, myofibroblasts are not found in normal cardiac tissue [4].

So far, no common definitive fibroblast-specific marker that could be used to identify fibroblasts in different tissues has been determined. In fact, fibroblasts in different tissues likely differ with respect to their cellular origins and functions. Several markers have been used to identify cardiac fibroblasts. Discoidin domain receptor 2 (DDR2) is specifically expressed by fibroblasts within the heart [20, 21], and, in a recent report, was successfully used as a marker in a flow cytometric analysis of cardiac cells [22]. This study found that fibroblasts represent a substantial portion of the cells in the mammalian heart. For example, the adult murine heart consists of approximately 55% cardiomyocytes and 45% nonmyocytes (~27% fibroblasts), and the adult rat heart consists of 30% cardiomyocytes and 70% nonmyocytes (~67% fibroblasts). Periostin [23] and thymus cell antigen-1 (Thy1/CD90) [14, 24] also have been used as markers of fibroblasts in developing and adult hearts. However, because fibroblasts can acquire heterogeneous phenotypes [25–27], those markers may not be capable of identifying all fibroblasts under all physiological and pathological conditions [5, 28]. Commonly used fibroblasts markers are reviewed elsewhere [28]. 

### 2.1. Origin and Phenotype of Cardiac Fibroblasts

**Figure 1:**
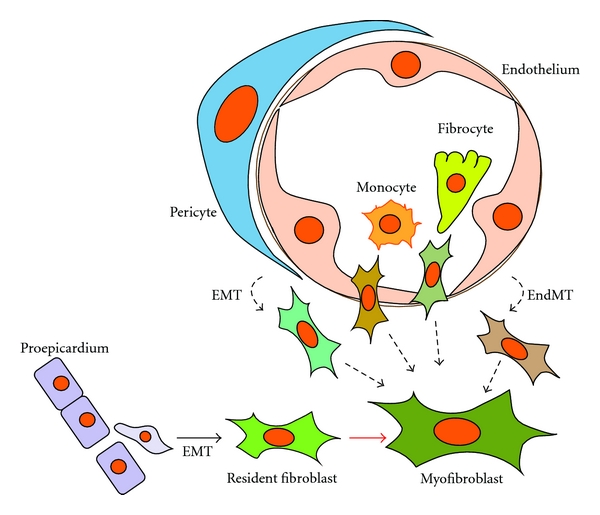
Diverse origins of cardiac fibroblasts. Resident cardiac fibroblasts are thought to arise from the proepicardium and embryonic epicardium during development. In response to fibrogenic stimuli, however, many other cell types, including bone marrow-derived cells, pericytes, and endothelial cells, may also acquire myofibroblast-like phenotypes. This scheme depicts the possible origins of cardiac fibroblasts. EMT: epithelial-to-mesenchymal transition; EndMT: endothelial-to-mesenchymal transition.

The majority of resident cardiac fibroblasts are thought to arise from embryonic proepicardial organ (Figure 1) [23, 29]. Proepicardial and primitive epicardial cells undergo epithelial-mesenchymal transition (EMT) and then migrate into the myocardium, where they progressively differentiate into interstitial fibroblasts, perivascular fibroblasts, and coronary SMCs [30, 31]. These resident fibroblasts have traditionally been thought to be the sole source of cardiac fibroblasts, but more recently other cellular origins of cardiac fibroblasts have been proposed [23]. Endothelial cells and pericytes may also contribute to cardiac fibroblasts via endothelial-to-mesenchymal transition (EndMT) and EMT, respectively [32, 33]. In addition, bone marrow-derived cells may acquire fibroblast-like phenotypes [32, 34–37], and fibrocytes, circulating mesenchymal progenitor cells of bone-marrow origin, have been shown to be recruited to ischemic hearts and to express SM *α*-actin, collagen I, vimentin, and DDR2 [38]. Finally, it has been suggested that monocytes/macrophages represent another potential source of myofibroblasts in ischemic hearts [39]. How these various bone-marrow-derived cells are related to one another remains unclear, as does their precise lineage origins, in part, because of a lack of definitive markers for fibroblasts and the different myeloid cell subsets. Macrophages may also promote fibrosis by producing cytokines, such as TGF-*β*. It is therefore likely that bone-marrow-derived cells play multiple roles in cardiac fibrosis. Further studies will be needed to clarify precisely how these cells contribute to cardiac fibrosis.

### 2.2. Mediators of Intercellular Communications in Fibrogenic and Cardiomyocyte Hypertrophic Responses

**Figure 2:**
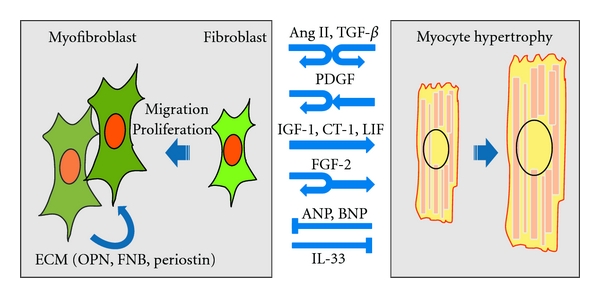
Reciprocal interactions between fibroblasts and cardiomyocytes. Many growth factors and cytokines have been shown to act in autocrine and/or paracrine fashion to induce hypertrophic responses in cardiomyocytes and activate fibroblasts. This scheme depicts only some of the factors identified. Ang II: angiotensin II; TGF-*β*: transforming growth factor-*β*; PDGF: platelet-derived growth factors; IGF-1: insulin-like growth factor-1; CT-1: cardiotrophin-1; LIF: leukemia inhibitory factor; FGF-2: fibroblast growth factor 2; ANP: atrial natriuretic peptide; BNP: brain natriuretic peptide; IL-33: interleukin-33; ECM: extracellular matrix; OPN: osteopontin; FBN: fibronectin.

Cardiomyocyte-specific deletion of genes has been shown to affect not only cardiomyocyte functionality but also the phenotypes and functions of fibroblasts [4]. Conversely, recent studies have shown that cardiac fibroblasts control cardiomyocyte proliferation in the developing ventricles during embryogenesis and that fibroblasts promote cardiomyocyte hypertrophy through paracrine factors and ECM [14, 24, 40]. These findings are indicative of the communication between cardiomyocytes and fibroblasts. Here we describe factors that mediate intercellular communication within the myocardium (Figure 2). 

#### 2.2.1. Angiotensin II

Ang II is a pleiotropic vasoactive peptide that plays key roles in the development of cardiac fibrosis and remodeling. Although most of the cardiovascular effects of Ang II are mediated via the Ang II type 1 receptor (AT1), the Ang II type 2 receptor (AT2) may be also important, as expression of both receptors is upregulated in various cardiac diseases [41–43]. Continuous infusion of Ang II into mice induces cardiac hypertrophy and fibrosis [44, 45]. Under these conditions, most of the proliferating fibroblasts were found to be surrounding cardiomyocytes carrying the AT1a receptor [46], suggesting that activation of cardiomyocytes via AT1a receptors also affects fibroblasts. Ang II also stimulates paracrine release of growth factors and cytokines, including TGF-*β*1 and endothelin-1 (ET-1) from cardiomyocytes [47–49]. On the other hand, AT1 receptor expression is greater in fibroblasts than cardiomyocytes [47], and Ang II directly stimulates fibroblast proliferation, collagen and ECM synthesis, and expression of fibroblast growth factor 2 (FGF2) [50]. It therefore seems likely that reciprocal interactions between cardiomyocytes and fibroblasts via paracrine factors are important for myocardial responses to Ang II. Consistent with these in vitro and in vivo findings in animal models are recent clinical studies demonstrating that blockade of the rennin-angiotensin system in patients, using a direct renin inhibitor, an angiotensin converting enzyme inhibitor, or an angiotensin receptor blocker, effectively reduces cardiac fibrosis and remodeling in addition to reducing blood pressure [51].

AT2 receptor expression is also upregulated in failing human hearts, mainly in cardiac fibroblasts [43, 52]; however, the function of AT2 remains controversial. Initially, AT2 was reported to mediate effects opposing the growth-promoting signals mediated by AT1 [53], but since then there have been several reports that AT2 also stimulates prohypertrophic signaling [54, 55]. The function of AT2 may depend on the adaptor proteins recruited to the receptor and the pathophysiological conditions [56, 57].

#### 2.2.2. Transforming Growth Factor-*β*

TGF-*β* exists in three isoforms (TGF-*β*1, TGF-*β*2, and TGF*β*-3) that have distinct but overlapping functions in immunity, inflammation, and tissue repair, and TGF-*β* also has a central role in fibroblast activation and differentiation into myofibroblasts [58]. TGF-*β* is initially produced as a latent complex bound to latent TGF-*β* binding protein (LTBP) within the interstitium. It is activated physiochemically by altered pH, a large group of proteases and enzymes, high-energy ionizing radiation, or integrin-mediated mechanisms [59, 60]. Activated TGF-*β* binds to heterodimers comprised of TGF-*β* type 1 receptor (TGF-*β*R1) and type 2 receptor (TGF-*β*R2) on both cardiomyocytes and fibroblasts [61]. TGF-*β*R1 (ALK5; activin-linked kinase 5) then phosphorylates receptor-regulated Smads (R-Smads: Smad2 and Smad3), which in turn associate with a common-mediator Smad (co-Smad: Smad4) and are translocated into the nucleus, where they act as transcription factors [62]. Smad3 is required for TGF-*β* to induce expression of collagen, fibronectin, and other ECM genes [63–66]. 

TGF-*β* promotes myofibroblast differentiation and ECM production by fibroblasts, and Ang II-induced cardiac hypertrophy is also mediated in part through TGF-*β* secreted from AT1-expressing fibroblasts [47]. TGF-*β*1-deficient mice subjected to chronic subpressor doses of Ang II showed no significant cardiac hypertrophy or fibrosis [67], which suggests that strategies to block TGF-*β* signaling may be useful for treating fibrogenic cardiac remodeling. Indeed, a TGF-*β* neutralizing antibody inhibited fibroblast activation and proliferation, and diastolic dysfunction in pressure-overloaded rats [68]. Similarly, an ALK5 inhibitor attenuated fibroblast activation and systolic dysfunction in an experimental rat model of myocardial infarction [69]. However, fibrosis was attenuated in Smad3-dificient mice subjected to experimental cardiac pressure overload, cardiac hypertrophy and heart failure were aggravated [70]. Moreover, TGF-*β* neutralizing antibody increased mortality and worsened cardiac remodeling, which correlated with reduction of ECM in a rat MI model [71]. These results indicate that the consequences of inhibiting TGF-*β* signaling can vary depending on the disease model and the timing of the inhibition, presumably because TGF-*β* signaling has an essential adaptive role in the myocardium under stress. Seemingly maladaptive functions, such as fibrosis, might also be essential for adaptation in other contexts. It will, therefore, be important to clarify the spatiotemporal functions of TGF-*β* signaling in different disease contexts if we are to develop effective therapeutic approaches involving TGF-*β*.

#### 2.2.3. Fibroblast Growth Factor-2

FGF-2 is alternatively translated as a high molecular weight (Hi-FGF-2) and a low molecular weight (Lo-FGF-2) isoform from the single *Fgf2* gene [72]. The Hi-FGF-2 isoform contains nuclear localization sequences and is found predominantly within the nucleus, while the Lo-FGF-2 isoform is localized in the ECM and cytoplasm [72]. Cardiac fibroblasts predominantly express Hi-FGF-2, which acts in a paracrine fashion to promote cardiomyocyte hypertrophy [49]. Hi-FGF-2 also acts in an autocrine fashion on the fibroblasts themselves to stimulate release of other pro-hypertrophic factors, such as cardiotrophin-1 (CT-1) [49, 73, 74]. In addition, Lo-FGF-2 elicits cardioprotective effects against postischemic cardiac dysfunction [72].

#### 2.2.4. Interleukins

The IL-6 family of cytokines, including leukemia inhibitory factor (LIF) and CT-1, are expressed by cardiac fibroblasts and cardiomyocytes. LIF and CT-1 secreted from fibroblasts mediate Ang II-induced cardiomyocyte hypertrophy [75, 76]. LIF was also shown to induce fibroblast hypertrophy but to inhibit myofibroblast transition and collagen deposition [77].

IL-33 is produced primarily by cardiac fibroblasts, and its expression is upregulated by cyclic strain [78]. IL-33 binds to a transmembrane form of the ST2 receptor (ST2L) on cardiomyocytes and inhibits the hypertrophic response of cultured cardiomyocytes to pro-hypertrophic stimuli. In vivo, IL-33 inhibits cardiomyocyte hypertrophy as well as fibrosis induced by pressure overload [40, 79].

#### 2.2.5. Serotonin

Serotonin (5-hydroxytryptamine [5-HT]) acts via its 5-HT_2B_ receptor (5-HT_2B_R) to contribute to cardiac hypertrophy. Indeed, plasma serotonin levels and cardiac 5-HT_2B_R expression are both elevated in human heart failure. 5-HT_2B_Rs mainly colocalize with AT1 receptors in fibroblasts [80, 81]. Isoproterenol (ISO) and Ang II-induced cardiac hypertrophy is suppressed in 5-HT_2B_R-deficient mice, and this effect is accompanied by reduced production of cytokines (IL-6, IL-1*β*, TGF-*β*, and TNF-*α*) and reactive oxygen species in cardiac fibroblasts [82, 83]. Similarly, pharmacological blockade of 5-HT_2B_Rs prevents ISO-induced murine cardiac hypertrophy [82]. Moreover, mice in which expression of 5-HT_2B_R is limited to their cardiomyocytes are also resistant to ISO-induced cardiac hypertrophy and dysfunction, as well as to ISO-induced upregulation of the cytokines. This suggests that signaling through 5-HT_2B_Rs on fibroblasts stimulates production of cytokines that promote cardiomyocyte hypertrophy [81, 84].

#### 2.2.6. Platelet-Derived Growth Factors

PDGF-A and -B are secreted from cardiomyocytes and fibroblasts and play critical roles in cardiac fibrosis and angiogenesis through their interactions with the protein tyrosine kinase receptors PDGF receptor (PDGFR)-*α* and -*β* [85, 86]. PDGF signaling activates fibroblast proliferation and migration and ECM deposition. PDGF expression is significantly increased in cardiac hypertrophy and fibrosis [87], atrial fibrillation [88], and chronic rejection of cardiac allografts [89]. PDGF-C and -D may also contribute to cardiac fibrosis and remodeling. Transgenic mice exhibiting cardiomyocyte-specific expression of PDGF-C and -D develop hyperproliferation of myocardial interstitial cells, resulting in progressive fibrosis leading to dilated cardiomyopathy and heart failure [90–92].

**Figure 3:**
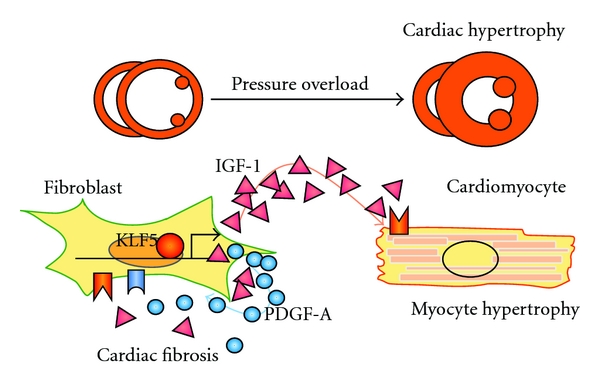
A model for the regulation of fibroblasts by KLF5 during cardiac hypertrophy. The transcription factor KLF5 controls *Igf1* and *Pdgfa* expression in cardiac fibroblasts. IGF-1 is a major cardiotrophic factor secreted from fibroblasts, and PDGF-A is primarily involved in mediating the migration and proliferation of fibroblasts.

PDGF signaling has been assessed as a therapeutic target for cardiac remodeling. The synthetic retinoid Am80 inhibited upregulation of PDGF-A by inhibiting the transcription factor Krüppel-like factor 5 (KLF5), thereby suppressing Ang II-induced cardiac fibrosis (Figure 3) [87, 93]. In addition, a neutralizing PDGFR*α*-specific antibody attenuated induction of pressure overload-induced atrial fibrosis and fibrillation [88]. However, several inhibitors of receptor tyrosine kinases, including PDGFRs, have been linked to the development of cardiomyopathy in some treated patients [94–97]. PDGFR-*β* on cardiomyocytes is indispensable for the cardiac response to pressure overload and may regulate angiogenesis [98]. For therapies targeting PDGF signaling to cardiac remodeling, it will be important to further clarify the precise roles played by PDGFs and PDGFRs under various pathological and physiological conditions. 

#### 2.2.7. Insulin-Like Growth Factor-1

IGF-1 exerts adaptive and cardioprotective effects in response to stress. The majority of serum IGF-1 is liver-derived and plays a critical role during normal body development. However, postnatal body growth is preserved, even in the complete absence of IGF-1 expression by hepatocytes; autocrine/paracrine IGF-1 appears to have important regulatory functions under these conditions [99]. In the heart, IGF-1 is mainly expressed in cardiac fibroblasts [14] and activates downstream signal transducers, such as phosphoinositide 3-kinase (PI3K), leading to cardiomyocyte hypertrophy [100]. We recently found that cardiac IGF-1 is transactivated by KLF5 (Figure 3) [14]. Although cardiac fibroblast-specific deletion of *Klf5* ameliorated the cardiac hypertrophy and fibrosis elicited by moderate-intensity pressure overload, it resulted in severe heart failure in high-intensity pressure overload. Similarly, administration of a peptide inhibitor of IGF-1 severely exacerbated heart failure induced by high-intensity pressure overload. These findings indicate that induction of IGF-1 is an essential cardioprotective response; that cardiac fibroblasts play a pivotal role in the myocardial adaptive response to pressure overload; that KLF5 controls IGF-1 expression in cardiac fibroblasts in response to stress [14].

#### 2.2.8. Connective Tissue Growth Factor

CTGF (also known as CCN2) is expressed in fibroblasts and cardiomyocytes and regulates ECM deposition and wound healing [101, 102]. CTGF is induced by TGF-*β*, Ang II, and ET-1. By itself, CTGF only weakly promotes fibrosis and cardiomyocyte hypertrophy, but it appears that it may promote a more robust effect by acting as a cofactor for TGF-*β* [103, 104]. Transgenic mice exhibiting cardiomyocyte-specific expression of CTGF did not develop cardiac hypertrophy or fibrosis under baseline conditions but showed significantly increased fibrosis and contractile dysfunction in response to pressure overload [105]. Another group of transgenic mice developed age-dependent cardiac hypertrophy and dysfunction, though Ang II did not increase fibrosis in young transgenic mice [106]. Thus, the cardiac actions of CTGF will require further study.

#### 2.2.9. Natriuretic Peptides

Atrial natriuretic peptide (ANP) and brain natriuretic peptide (BNP) are usually synthesized in the atria and ventricles, respectively [107]. Plasma levels of these peptide hormones are widely used as biomarkers when making a diagnosis or determining risk stratification in a variety of cardiac disease states. In addition, infusion of synthetic ANP or BNP is useful for treating cardiac heart failure and remodeling, mainly by optimizing intravascular volume and arterial pressure [108]. ANP and BNP also exert anti-hypertrophic and antifibrogenic effects on the heart, and knockout mice deficient in their common receptor, guanylyl cyclase-A (GC-A), showed cardiac hypertrophy and extensive interstitial fibrosis that was at least partially independent of blood pressure [109–111]. In fibroblasts, BNP inhibits TGF-*β*-regulated genes related to fibrosis (collagen I, fibronectin, and CTGF), proliferation (PDGF-A and IGF-1), and inflammation (COX2, IL-6, and TNF) [112], while ANP suppresses ET-1 expression and cell proliferation [113]. This suggests ANP and BNP secreted from cardiomyocytes suppress the fibrogenic activity of fibroblasts.

#### 2.2.10. ECM Molecules

ECM serves as an important intermediary for intercellular communication by transducing intracellular signals via its receptor molecules (integrins and CD44) on myocardial cells [24, 114–116]. Production, degradation and modification of ECM components are dynamically regulated under both physiological and pathological conditions. Fibroblasts are a major source of nonbasement membrane collagen and other ECM proteins, and other cells in the myocardium, including cardiomyocytes, endothelial cells, and SMCs, also produce sets of ECM components [117]. Cardiac fibroblasts and macrophages are major producers of matrix metalloproteases (MMPs), which degrade ECM proteins.

The integrin family consists of 18 *α* and 8 *β* subunits, which form 24 known *α*-*β*-heterodimers. Integrins serve as cell-ECM and cell-cell adhesion molecules and also function as signal-transducing receptors for ECM proteins, including collagen, laminin, fibronectin (FBN), and osteopontin (OPN) [116, 118]. Cardiac-specific integrin signaling in genetically engineered animal models are reviewed elsewhere [116].

CD44 was originally described as a receptor for hyaluronan (HA), a ubiquitous constituent of the ECM, but is now known to interact with collagen, laminin, FBN, and OPN [119]. CD44-HA interactions play an important role in regulating leukocyte extravasation into sites of inflammation and in mediating efficient phagocytosis. CD44 also contributes to the resolution of inflammation through removal of matrix breakdown products, clearance of apoptotic neutrophils, and fibroblast migration [120]. In injured hearts, CD44 is upregulated in fibroblasts, leukocytes, and endothelial cells, particularly those cells surrounding and within the coronary arteries [120, 121]. *Cd44*-deficient mice subjected to myocardial infarction show increased myocardial infiltration by leukocytes and expression of proinflammatory cytokines, followed by decreased fibroblast infiltration and fibrosis and enhanced dilative cardiac remodeling [120]. Finally, *Cd44*-deficient cardiac fibroblasts exhibit diminished proliferation and collagen synthesis in response to TGF-*β*. This suggests CD44 is important for resolution of postinfarction inflammatory processes and for regulation of fibroblast function. 

FBN and OPN are upregulated in cardiac hypertrophy and by Ang II. They contain the arginine-glycine-aspartate (RGD) tripeptide integrin binding motif and activate integrin-mediated proliferation, survival, adhesion, differentiation, and migration of myocardial cells [122–124]. FBN is mainly expressed in fibroblasts and acts in a paracrine fashion to regulate cardiomyocyte proliferation through *β*1 integrin signaling during embryonic heart development [24].

OPN is strongly expressed in chronic inflammatory and autoimmune diseases and promotes the recruitment and retention of macrophages and T cells at inflamed sites [125]. Cardiomyocyte-specific overexpression of OPN results in dilated cardiomyopathy and severe fibrosis, with recruitment of activated T cells showing Th1 polarization [125]. Moreover, OPN (*Spp1*)-deficient fibroblasts are less proliferative and less adherent to ECM substrates, while *Spp1*-deficient mice exhibit less Ang II-induced cardiac fibrosis [123, 126]. Recently, OPN has emerged as a novel biomarker of various cardiac diseases [127–129].

Periostin is primarily expressed in myocardial fibroblasts, and its expression is upregulated by pressure overload and myocardial infarction. Periostin can serve as a ligand for *α*v*β*3, *α*v*β*5, and *α*4*β*6 integrins [23] and can also directly interact with other ECM proteins, including FBN, tenascin-C, collagen I/V, and heparin [130]. Collagen fibrils from periostin (*Postn*)-null mice are reduced in size, somewhat disorganized, and less efficiently cross-linked, indicating that periostin facilitates proper organization of the ECM [23, 131, 132]. In addition, periostin is induced via TGF-*β* signaling and may then enable collagen realignment in response to TGF-*β* [130, 133, 134]. *Postn*-deficient hearts subjected to pressure overload or ischemic insult exhibited less fibrosis but more frequent rupture of the ventricular wall [132, 135].

## 3. Endothelial Cells

Vascular endothelial cells are also crucially involved in the development of cardiac hypertrophy, remodeling, and failure. Endothelial cells are capable of producing a wide variety of functional agonists and antagonists, including vasodilators and vasoconstrictors, procoagulants and anticoagulants, and inflammatory and anti-inflammatory factors. Endothelial cells maintain homeostasis by controlling the balance of these various mediators [136]; endothelial dysfunction disturbs that balance and leads to pathological inflammatory processes. For instance, activated endothelial cells express the adhesion molecules, intercellular adhesion molecule-1 (ICAM-1) and vascular cell adhesion molecule-1 (VCAM-1), which recruit and promote the infiltration of immune cells into the myocardium in response to various stimuli.

 Endothelial cell-derived nitric oxide (NO), produced by endothelial NO synthase (eNOS), is a key regulator of vasodilation. NO also reduces vascular permeability and thrombogenesis, and it inhibits inflammation by suppressing signaling by adhesion molecules, proinflammatory cytokines, and NF-*κ*B [136]. Under pathological conditions, the bioavailability of NO is diminished [137, 138]. For example, sustained pressure overload triggers eNOS uncoupling, which reduces NO signaling, increases levels of eNOS-derived reactive oxygen species, and promotes endothelial dysfunction. NO is also known to influence myocardial excitation-contraction coupling, substrate metabolism, and hypertrophy, as well as cell survival, which are at least in part dependent on eNOS and nNOS expression in cardiomyocytes [139]. For example, eNOS (*Nos3*) knockout mice develop concentric left ventricular hypertrophy and fibrosis [140], indicating the importance of the autocrine and paracrine effects of NO in cardiac remodeling.

Accumulating evidence indicates that impaired angiogenesis contributes to the transition of cardiac hypertrophy to heart failure. Hypertrophic stimuli induce expression of the angiogenic growth factors, vascular endothelial growth factor (VEGF), and angiopoietin 2 [141], which promote angiogenesis and blood flow in response to reductions in coronary perfusion pressure or ischemia. Blockade of VEGF action using an adenoviral vector encoding a decoy VEGF receptor or an anti-VEGF antibody promotes the transition from compensatory cardiac hypertrophy to failure in response to pressure overload in mice [142, 143]. Likewise, TNP-470, an inhibitor of angiogenesis, also induced cardiac dysfunction [144]. Conversely, VEGF treatment during prolonged pressure overload preserved contractile function [144, 145].

Within myocardium subjected to pressure overload, hypoxia-inducible factor-1-(HIF-1-) mediated transactivation of VEGF in cardiomyocytes plays an important role during induction of angiogenesis. Furthermore, it has been proposed that, in response to sustained pressure overload, p53 accumulates in cardiomyocytes and inhibits HIF-1 activity, thereby impairing cardiac angiogenesis and contractile function [144]. However, there are also conflicting data showing that ventricular deletion of HIF-1*α* prevents hypertrophy-induced activation of peroxisome proliferator-activated receptor-(PPAR-) *γ* and contractile dysfunction [146].

Endothelin-1 (ET-1) is a major growth factor secreted from endothelial cells that contributes to cardiac hypertrophy and fibrosis. ET-1 was originally identified as an endothelium-derived vasoconstrictor [147], but it is also expressed in various nonendothelial cells, including fibroblasts and cardiomyocytes, and it exerts both autocrine and paracrine effects that appear to be important for cardiomyocyte hypertrophy [47, 48, 148]. Although cardiomyocytes predominantly express ET_A_ ET-1 receptors, the hypertrophic response to Ang II and ISO is unaltered in cardiomyocyte-specific ET_A_ receptor-(*Ednra-*) deficient mice, suggesting the possible involvement of ET_A_ receptors on nonmyocytes and ET_B_ receptors in cardiomyocyte hypertrophy [48, 149]. Consistent with that idea, the combined ET_A_/ET_B_ receptor antagonist bosentan inhibited Ang II-induced cardiac hypertrophy [150]. It was also shown that endothelium-derived ET-1 promotes cardiac fibrosis in diabetic heart [151].

## 4. Immune Cells

A variety of immune cells, including macrophages, T cells, and mast cells, reside in the myocardium under physiological conditions. They are also induced to infiltrate the myocardium under pathogenic conditions and to promote cardiac remodeling, in part by releasing cytokines, growth factors, and MMPs [152, 153].

Macrophages are essential effector cells involved in tissue remodeling and fibrosis. It is becoming increasingly clear that macrophages can have diverse phenotypes and functions [154]. In vitro studies have shown that Th1 cytokines, alone or in contact with microbial products, elicit classical M1 activation of macrophages, which then release proinflammatory cytokines and reactive oxygen species. Th2 cytokines (IL-4 and IL-13) elicit an alternative form of macrophage activation designated M2. M2 macrophages are thought to suppress immune responses and promote fibrosis and tissue remodeling, though M2 activation is a rather generic term often used to describe any form of macrophage activation other than classical M1. Previous studies have shown that macrophages are involved in cardiac hypertrophy and remodeling. For instance, Ang II-induced cardiac hypertrophy and fibrosis were diminished in macrophage-specific mineralocorticoid receptor-(MR-) deficient mice [155]. The MR-deficient macrophages exhibited M2-type activation and reduced expression of proinflammatory cytokines, suggesting it is M1-type macrophages that are involved in the cardiac hypertrophy and fibrosis induced by Ang II. Similarly, a monoclonal neutralizing anti-MCP-1 antibody attenuated not only macrophage accumulation, but also fibroblast proliferation and fibrosis, resulting in amelioration of cardiac diastolic dysfunction [156]. These results demonstrate the pathological involvement of macrophages in cardiac hypertrophy and fibrosis. By contrast, macrophage depletion using liposomal clodronate induces abundant infiltration of inflammatory cells, predominantly CD4^+^ lymphocytes, and aggravates cardiac dysfunction in hypertensive rats harboring the mouse renin gene (*Ren2*) [157]. This suggests macrophages exert a protective effect against cardiac dysfunction induced by hypertension. Clearly further studies are needed to clarify the seemingly diverse functions of macrophages in cardiac hypertrophy and heart failure. It is very likely that macrophage function changes with time and in different pathological contexts [158]. It will therefore be important to characterize the different functions and phenotypes of macrophages at different times during the processes of cardiac hypertrophy and heart failure and to elucidate the underlying molecular mechanisms. These studies will be essential for the development of novel therapeutic interventions affecting macrophages.

Mast cells reside in the myocardium, and their numbers are increased in hypertrophied and failing hearts. Mast cells are an important source of an array of cytokines, growth factors, chemokines, and other mediators. Histamine is a major mediator released upon mast cell degranulation in the heart and may be involved in heart failure. Consistent with that idea, inhibition of histamine using the histamine H_2_ receptor antagonist famotidine reportedly ameliorates heart failure [159, 160]. In addition, mast cells secrete the proteases renin and chymase, which, respectively, catalyze the conversion of angiotensinogen to angiotensin I and angiotensin I to Ang II and may thus activate the local rennin-angiotensin system in the heart [161]. Mast cell degranulation also releases preformed TGF-*β*, PDGF-A, and TNF-*α* [88, 161, 162], and inhibition of mast cells suppresses cardiac dysfunction and atrial fibrillation induced by pressure and volume overload [88, 163]. These results strongly suggest that mast cells are involved in inflammatory processes that contribute to remodeling in the heart.

T cells also reside in the myocardium. Although little is known about their function in cardiac pathology, Kvakan et al. recently reported that adoptive transfer of CD4^+^ CD25^+^ regulatory T cells suppresses cardiac hypertrophy and fibrosis induced by Ang II in mice, and this effect was accompanied by a marked reduction in infiltration of inflammatory cells [164].

## 5. Cardiac Progenitor Cells

In addition to fibroblasts and immune cells, the cardiac stroma contains a number of mesenchymal cell types. Previous studies have demonstrated that a fraction of these mesenchymal cells, referred to as cardiac progenitor cells (CPCs), have the potential to differentiate into cardiomyocytes under certain conditions [165]. There is also some evidence of cellular interactions between CPCs and their surrounding cells, including cardiomyocytes and fibroblasts. For instance, IGF-1 and Ang II produced by surrounding cells affect the survival of CPCs [166, 167]. In addition, human CPCs were shown to be connected to cardiomyocytes and fibroblasts via gap junctions and adherens junctions [168, 169], which may enable cellular communication via mediating molecules, including miRNA [170]. Communication between CPCs and surrounding cells through Notch-Notch ligand and Eph-ephrin signaling has also been shown [171, 172]. As such, cellular communication between mesenchymal cells, including CPCs, and their surrounding cells, including cardiomyocytes and fibroblasts, may contribute to the myocardial response to injury.

## 6. Conclusions

Both cardiomyocytes and nonmyocytes play essential roles in the processes involved in the development of cardiac hypertrophy, remodeling, and failure. The cellular and molecular processes that contribute to cardiac remodeling and failure share many features with chronic inflammatory processes in other tissues. Thus sterile stresses such as pressure overload and Ang II appear to activate pathways that are commonly used in inflammatory processes, including those involving immune cells in the myocardium. As we have seen here, dynamic cellular interactions among cardiomyocytes and nonmyocytes are a driving force for these inflammatory processes. In that regard, it will be important to further clarify the functional involvement of the different cell types residing in the myocardium and the underlying molecular control mechanisms. The function of a particular cell type may change with time and in response to different insults. As discussed, the functions of some myocardial cells, such as fibroblasts and macrophages, appear maladaptive under certain conditions but are in fact essential for adaptive responses at different times and in different disease models. Elucidation of these complex processes could lead to identification of novel therapeutic targets for the treatment of cardiac hypertrophy and heart failure.
